# Exploring the relationship between boredom proneness and short-form videos addiction among Chinese college students through a moderated mediation model

**DOI:** 10.1038/s41598-025-22313-7

**Published:** 2025-11-03

**Authors:** Zihao Wan, Mengru Guo, Chi Yang, Wenqing Li, Xiaoyu Li, Wang Zheng, Yinqiu Zhao

**Affiliations:** 1 Faculty for Physical Education, Shanghai International Studies University, Shanghai, 201620 China; 2 College of Literature and Law, Henan Agricultural University, Zhengzhou, 450002 China; 3 School of Psychology, South China Normal University, Guangzhou, 510631 China; 4 School of Management, Jinan University, Guangzhou, 510632 China; 5 The School of Psychology and Cognitive Science, East China Normal University, Shanghai, 200062 China

**Keywords:** Short-form video addiction, Boredom proneness, Fear of missing out, Intolerance of uncertainty, College students, Psychology, Human behaviour

## Abstract

Previous studies have shown positive associations between boredom proneness and mobile phone addiction. However, the association between boredom proneness and short-form video addiction (SFVA), as well as the intermediate psychological mechanisms, remain unclear. This study aimed to investigate the correlation between boredom proneness and subsequent SFVA among Chinese college students and to examine the mediating role of fear of missing out and the moderating role of intolerance of uncertainty. A total of 458 college students (62.2% male; *M*_age_ = 19.17) from a university in central China participated in a two-wave time-lagged study with six months intervals. Results showed that boredom proneness was positively associated with SFVA six months later. Fear of missing out mediated this association, and intolerance of uncertainty moderated the association between boredom proneness and fear of missing out. The results further showed that the indirect effect of boredom proneness on SFVA via fear of missing out was more salient when there was a higher levels of intolerance of uncertainty. Interventions targeting boredom proneness and intolerance of uncertainty may be important in addressing SFVA in college populations.

## Introduction

The emergence of short-form video platforms, epitomized by *TikTok*, offers users the ability to edit, upload, and share short-form videos while delivering highly personalized content by analyzing user browsing behaviors, preferences, and interaction data, making it especially popular among youth^1^. As a fast-paced, shareable and easily accessible form of entertainment, short-form videos not only greatly satisfy college students’ entertainment, expression and social needs, but also increase the risk of them becoming addicted to short-form video platforms^2^. Internet addiction can be classified into two types: broad internet addiction and specific internet addiction. Broad internet addiction encompasses general or multi-dimensional excessive internet usage behavior, while specific internet addiction refers to specific types of internet dependence with similar characteristics, such as gaming addiction, social media addiction, and short-form video addiction, etc^3^. Unlike other forms of specific internet addiction that typically target a single psychological need, short-form video platforms are more conducive to addictive behavior because they simultaneously fulfill the desire for convenience, entertainment, and social connection^4^. Within this framework, short-form video addiction (SFVA) is defined as an individual’s inability to self-regulate their use of short-form video applications, despite significant distress and functional impairment, which is thus considered a specific subtype of internet addiction^2^. As with other subtypes of internet addiction, SFVA is often associated with a range of negative psychological and physiological outcomes, including emotional dysregulation^5^ and sleep disturbances^6^. Moreover, unlike more stable subtypes of internet addiction, SFVA is more likely to impair sustained attention and reduce information-processing efficiency, and displays a rapidly accelerating growth^7,8^, indicating that its etiology and underlying addiction mechanisms may differ from other forms of addictive behavior. Therefore, given the increasing prevalence of short-form videos and their adverse effects on youth, researchers urgently need to investigate the potential risk factors associated with SFVA.

Nevertheless, several important gaps remain in this line of research. First, recent research has demonstrated that boredom proneness is a significant individual trait strongly associated with SFVA^9^. However, there is limited research exploring the intermediate mechanisms between boredom proneness and SFVA. Most studies consider boredom proneness as a mediating variable rather than an independent variable^9–11^. Second, the research mainly focuses on general internet addiction or mobile phone addiction, while there is a lack of exploration regarding the uniqueness of SFVA^12,13^.Thus the present study aims to examine the effect of boredom proneness on SFVA through a time-lagged design, with fear of missing out (FoMO) as a mediator and intolerance of uncertainty as a moderator, in order to clarify its underlying mechanisms.

### Boredom proneness and short-form video addiction

Boredom proneness is an individual trait that reflects a person’s susceptibility to experiencing boredom across various contexts^14^. Research indicates that between 2009 and 2020, the popularity of the internet and smartphones has significantly increased the tendency of Chinese college students to get bored^15^. An Interaction of Person–Affect–Cognition–Execution (I-PACE) model^16^ underscores the pivotal role of personal traits (e.g., boredom proneness) in the development of specific Internet use disorders or overdependence. Within the model’s framework, boredom is characterized as a negative affective experience or mental state, defined by a desire for meaningful or engaging experiences^17^. Whereas individuals with high levels of boredom proneness are more likely to experience boredom and loneliness in low-stimulus environments (e.g., college campuses)^18^ because they struggle to focus during extended periods of monotony and lack the intrinsic motivation to seek out new social opportunities. To seek emotional comfort in social relationships and fulfill entertainment needs, online short-form video platforms may serve as “emotional sustenance” for bored college students^19^. Furthermore, the compensatory internet use theory suggests that individuals engage in online activities to alleviate negative affect or compensate for unmet psychological needs^20^. From this perspective, boredom proneness represents a key vulnerability factor, as it reflects chronic difficulty in generating intrinsic stimulation and a tendency to perceive environments as unstimulating^21^. Short-form video platforms, with their algorithm-driven personalization and immediate reward mechanisms, provide an ideal outlet for compensating such deficits^2^.

Previous research on mobile phone addiction provides some evidence of the predictive effects of boredom proneness on SFVA. For instance, two other cross-sectional study of Chinese university students revealed a significant positive association between boredom proneness and smartphone addiction^11,22^. One lonitudinal study of Chinese college students has shown that boredom proneness was associated with mobile phone addiction 8 months later^13^. Moreover, one meta-analysis of 59 empirical studies showed a medium-to-large positive association between boredom and problematic digital media use^23^. A recent review of 28 studies found that an individual’s boredom trait is directly related to problematic technology use (such as excessive use of smartphones and social media), and it also acts indirectly through intermediary variables^24^. Furthermore, a recent cross-sectional study involving 361 adolescents found that the perception of leisure boredom among teenagers has an indirect influence on the addiction to short-form videos^25^, Another longitudinal study conducted with 699 Chinese university students indicated a reciprocal relationship between boredom proneness and problematic online video watching^10^. However, most existing studies are cross-sectional and have primarily examined general internet or smartphone addiction, with limited attention to specific subtypes such as SFVA. In addition, prior research often conceptualizes boredom proneness as a mediating variable rather than as an independent predictor. Addressing these gaps, the present study adopts a time-lagged design to examine boredom proneness as a direct antecedent of SFVA, thereby extending previous findings and providing new insights into the unique mechanisms underlying this specific subtype of internet addiction.

### The mediating effect of fear of missing out

Fear of Missing Out (FoMO) is defined as a pervasive anxiety that others may be engaging in rewarding experiences in one’s absence^26^. This emotion is expressed as a concern about missing out on social media messages and a constant focus on the activities that others are engaging in. According to the I-PACE model^16^, FoMO may serve as a critical mediator between boredom proneness and SFVA. In the context of the Internet’s capacity to offer an ever-increasing amount of social content and information, individuals with high boredom proneness are likelier to seek meaningful and stimulating content online. This reliance on external stimuli heightens their anxiety about missing potentially engaging information, making them more eager to maintain constant social contact^27^. Consequently, this heightened anxiety and unease about missing out on social opportunities or significant information (i.e., FoMO) may emerge as a natural extension and emotional response to the experience of boredom. In turn, this compels individuals to increasingly engage in Internet activities (e.g., short-form video use) as a means of temporarily alleviating tension, fear, and anxiety. Although this form of emotional regulation may offer short-term relief, it concurrently intensifies their dependence on short-form video platforms, ultimately increasing the risk of developing SFVA.

Existing research provides partial support for the mediating role of FoMO in the relationship between boredom proneness and SFVA. For instance, a cross-sectional study of U.S. adults found that boredom proneness positively associated with FoMO^28^, which was also supported in another cross-sectional study of U.S. college students^29^. Furthermore, numerous studies have supported the relations between FoMO and SFVA. For example, a meta-analysis comprising 86 studies and 55,134 participants found a significant association between FoMO and excessive internet use^30^. Another cross-sectional study of 2,744 Chinese adolescents and young adults demonstrated that FoMO significantly associated with social media addiction, particularly when the desire to express oneself was high and others frequently viewed messages^31^. More importantly, a study conducted with a sample of 297 American college students revealed a significant positive association between boredom proneness and problematic smartphone use, with FoMO serving as a critical mediator in this relationship^29^. This finding suggests that FoMO is not merely a risk mechanism specific to problematic smartphone use but may also constitute a pivotal psychological pathway through which boredom proneness contributes to broader forms of technology-related addictive behaviors, such as SFVA. Although prior research has established robust links between boredom proneness and both smartphone and internet use disorders, the mediating role of FoMO in the context of SFVA has yet to be systematically examined.

### The moderating effect of intolerance of uncertainty

The experience of boredom is typically associated with a lack of social interaction and a strong desire for novel stimuli, indicating an absence of effective information and engagement^17^. Intolerance of uncertainty, defined as an aversive reaction to insufficient or incomplete information^32^, may be an important risk factor that amplifies the negative impact of boredom,

individuals with high intolerance of uncertainty tend to perceive uncertain events as threatening and distressing, regardless of the actual likelihood of their occurrence. Drawing on the I-PACE model^16^, boredom proneness can be conceptualized as an affective vulnerability that increases individuals’ need for external stimulation, whereas intolerance of uncertainty represents a cognitive disposition reflecting low tolerance for ambiguity. Similarly, individuals with higher boredom proneness tend to experience difficulty in sustaining stable sources of stimulation, which heightens their sensitivity to novel or uncertain situations. When intolerance of uncertainty is also high, this sensitivity may be amplified, leading individuals to perceive potential social or recreational opportunities as more salient and pressing, thereby intensifying FoMO. In such cases, individuals are more likely to turn to short-form video platforms, to alleviate the resulting anxiety, worry, and unease, thereby further intensifying their reliance on these platforms.

Although there is a lack of direct evidence that intolerance of uncertainty strengthens the link between boredom proneness and FoMO, existing research indicates that individuals with high levels of intolerance of uncertainty are more prone to experiencing negative emotions, including anxiety, worry, and fear in response to negative life events. For example, a study of Australian undergraduates found that individuals with high levels of Intolerance of uncertainty exhibited stronger worry and negative emotional reactions to uncertain situations^33^. Another longitudinal study of Canadian undergraduates found that individuals with high intolerance of uncertainty experienced a more significant increase in negative affect in response to daily distress compared to those with low intolerance of uncertainty^34^. Another cross-sectional study of 1,092 U.S. college students observed that Intolerance of uncertainty significantly moderated the relationship between daily stress and worry. Specifically, individuals with high intolerance of uncertainty were more likely to exhibit inhibitory responses to daily stress compared to those with low intolerance of uncertainty, which subsequently led to increased experiences of worry and anxiety^35^. Therefore, intolerance of uncertainty may play as a crucial role of emotion regulation in the context of boredom and information deprivation. intolerance of uncertainty not only amplifies an individual’s negative emotional responses but also compels them to seek immediate external stimuli to alleviate psychological distress. This contradiction between short-term emotional relief and long-term dependence can easily lead individuals to develop continuous information cravings and behavioral addictions, such as SFVA.

### The current study

**Fig. 1:**
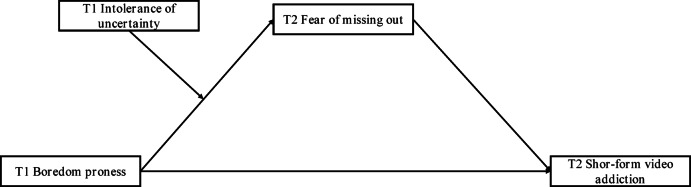
Hypothesized moderated mediation model from boredom proneness to short-form video addiction; T1 = Time 1; T2 = Time 2.

Numerous studies have demonstrated a strong association between boredom proneness and problematic smartphone and internet use among college students^11,22,36^. However, most prior studies were cross-sectional and did not adequately address the potential intermediate mechanisms involved in these associations. Therefore, this study aimed to explore the relationships among boredom proneness, fear of missing out (FoMO), intolerance of uncertainty, and SFVA (See Fig. 1). Based on existing literature, this study proposed the following three hypotheses: (1) boredom proneness is positively associated with SFVA among college students; (2) FoMO mediates the association between boredom proneness and SFVA; (3) the association between boredom proneness and FoMO is stronger among individuals with high intolerance of uncertainty, leading to higher levels of SFVA.

## Methods

### Participants and procedure

Convenience sampling was utilized in this study to recruit college students from one university in central China. The study followed a two-wave design to track participants over time. The initial survey (Time 1; T1) was conducted in May 2023, collecting baseline data on key variables. Six months later (Time 2; T2), participants who completed the T1 survey were invited to participate in a follow-up survey to assess changes over time. The measurement timeline was based on two key frameworks: the I-PACE model^16^ for specific internet use disorders and the requirements for inferring mediation in time-series research^37^. The I-PACE model posits that relatively stable personal factors predispose individuals to subsequent, more immediate affective-cognitive mechanisms, which in turn facilitate addictive behaviors. Over time, conditioned reinforcement strengthens these associations. To align with this temporal sequence, personal factors (i.e., boredom proneness, intolerance of uncertainty) were measured at T1, while proximal mechanisms (i.e., FoMO) and behavioral outcomes (i.e., SFVA) were measured at T2. This data collection sequence aligns with the temporal priority assumptions of the I-PACE model and maximizes avoidance of the inevitable reverse causality issues and common method bias inherent in simultaneous measurement of all variables^38,39^. After consent, each participant was assigned a unique alphanumeric code used solely to link waves across time. Questionnaires were completed via a secure electronic platform, so teachers and classmates could not access responses. Identifiable contact information was only access restricted to the data manager. Respondents who gave consent to participate proceeded through a series of online questionnaires in regular classrooms by two trained graduate assistants employing identical verbal and written instructions. The participants were allowed to take as much time as necessary to complete the measures and had the option to withdraw from the study at any time. The study was approved by Human Research Ethics Committee of South China Normal University (Project number: SCNU-PSY-2023-417). Researchers explained the basic nature of the study to students and assured them that their responses would be strictly confidential.

A total of 624 college students took part in this study at T1. Among these students, 458 completed the online survey at T2. Attrition was mainly due to students failing the attention check, unwillingness to participate, or absence from school on the day in the second measurement. Attrition analysis was conducted to examine potential bias between participants who had completed measures across all two time points (Group 1) and participants who dropped out at T2 (Group 2). Results indicated that the two groups did not differ in gender (*χ*^2^ (d*f*) = 2.632(1), *p* = 0.105), age (*χ*^2^ (d*f*) = 7.063(3), *p* = 0.070), mother’s education level (*χ*^2^ (d*f*) = 9.425(5), *p* = 0.093), family monthly income per personl (*χ*^2^ (d*f*) = 8.091(5), *p* = 0.151), T1 boredom proneness (*t*(d*f*) = −0.457(622), *p* = 0.647), and T1 intolerance of uncertainty (*t*(d*f*) = 0.591(622), *p* = 0.555), but differed in father’s education level (*χ*^2^ (d*f*) = 11.395(4), *p* < 0.05). Given that participants with missing T2 data could not be included in the complete data analysis, only students who participated fully in both assessments (*N* = 458; 62.2% male; *M*_age_ = 19.17, SD = 0.80) were included in the present study. In the final sample, 61.8% of fathers and 71.0% of mothers had a “junior high school or below” education; 29.0% of fathers and 19.4% of mothers had a “senior high school or vocational school” education; and 9.2% of fathers and 9.6% of mothers held a “bachelor’s degree or above.” Regarding family monthly income per person, 6.1% of participants reported an income under 500 yuan, 17.5% reported between 500 and 2000 yuan, 34.9% reported between 2000 and 5000 yuan, and 41.5% reported more than 5000 yuan.

### Measures

#### Boredom proneness

The Boredom Proneness Scale-Short Form (BPS-SF^14^;; Chinese version^40^: was used to measure the tendency to experience boredom. It consists of 12 items (e.g., “I was often in monotonous and tiresome situations.”). Ratings for items are based on a 7-point Likert scale, ranging from 1 (completely disagree) to 7 (completely agree). Higher mean scores indicate higher level of boredom proneness. In this study, the Cronbach’s alpha coefficients for the BPS-SF at T1 was 0.83. The results of the confirmatory factor analysis indicate that the scale has a good structure in this study: *χ*^2^/d*f* = 2.916, CFI = 0.980, TLI = 0.936, RMSEA = 0.065, SRMR = 0.032.

#### Fear of missing out

The Fear of Missing Out Scale (FoMOS^26^;; Chinese version^41^: was used to measure the level of fear of missing out. It consists of 10 items (e.g., “I fear others have more rewarding experiences than me.”). Ratings for items are based on a 5-point Likert scale, ranging from 1 (not at all true of me) to 5 (extremely true of me). Higher mean scores indicate higher levels of FoMO. In this study, the Cronbach’s alpha coefficients for the FoMOS at T2 was 0.94. The results of the confirmatory factor analysis indicate that the scale has a good structure in this study: *χ*^2^/d*f* = 3.427, CFI = 0.987, TLI = 0.974, RMSEA = 0.073, SRMR = 0.031.

#### Short-form video addiction

The Chinese version of Short-form Video Addiction Scale^42^ revised from Young’s Internet Addiction Diagnostic Questionnaire^43^ was used to assess SFVA. It consists of 8 items (e.g., “Have you repeatedly made unsuccessful efforts to control, cut back, or stop short-form video use?”). Ratings for items are based on a 5-point Likert scale, ranging from 1 (completely inconsistent) to 5 (completely consistent). Higher total scores indicate higher levels of SFVA. In this study, the Cronbach’s alpha coefficients for the SFVA at T2 was 0.94. The results of the confirmatory factor analysis indicate that the scale has a good structure in this study: *χ*^2^/d*f* = 4.944, CFI = 0.988, TLI = 0.968, RMSEA = 0.076, SRMR = 0.019.

#### Intolerance of uncertainty

The short form of the Intolerance of Uncertainty Scale (IUS-12^44^;; Chinese version^45^: was utilized in this study.It consists of 12 items (e.g., “It frustrates me not having all the information I need.”). Ratings for items are based on a 5-point Likert scale, ranging from 1 (not at all characteristic of me) to 5 (entirely characteristic of me). Higher mean scores indicate lower levels of tolerance of uncertainty. In this study, the Cronbach’s alpha coefficients for the IUS-12 at T2 was 0.92. The results of the confirmatory factor analysis indicate that the scale has a good structure in this study: *χ*^2^/d*f* = 3.633, CFI = 0.970, TLI = 0.952, RMSEA = 0.076, SRMR = 0.033.

#### Covariates

College students’ age, gender, parental education levels, and family monthly income per person were reported by the students at T1. Parental education levels were assessed on a scale ranging from 1 (*Junior high school and below*) to 6 (*doctoral degree*) and average monthly family income were also assessed on a scale ranging from 1 (*500¥ and below*) to 6 (*10*,*000¥ and above*).

### Statistical analyses

Firstly, the current study proceeded with descriptive and correlation analyses concerning the variables in SPSS 22.0. Secondly, Model 4 and Model 7 of PROCESS 4.0^46^ were executed to examine the mediating role of FoMO in the relation between boredom proneness and SFVA and the moderating role of intolerance of uncertainty in the relation between boredom proneness and FoMO. Furthermore, 95% bootstrap confidence intervals were computed based on 5000 bootstrapped samples to enhance the robustness of the findings, with age, gender, parental education level, and average monthly family income as covariates.

## Results

### Preliminary analyses

As shown in Table 1, correlations among boredom proneness, fear of missing out, intolerance of uncertainty, and short-form video addiction were all positive and statistically significant. Therefore, the results of the correlation analysis preliminarily support the hypotheses of this study, namely, the longitudinal association between boredom proneness and SFVA, the mediating role of FoMO, and the moderating role of intolerance of uncertainty.

**Table 1:** Descriptive statistics and correlations for study variables (*n* = 458).

	1	2	3	4	5	6	7	8	9
1. Age	-								
2. Gender	− 0.03	-							
3. Father’s education level	0.03	− 0.14^**^	-						
4. Mother’s education level	0.03	− 0.15^**^	0.58^***^	-					
5. Average monthly family income	− 0.03	− 0.09	0.20^***^	0.23^***^	-				
6. T1 Boredom proneness	− 0.11^*^	− 0.03	0.01	− 0.02	− 0.07	-			
7. T1 Intolerance of uncertainty	0.05	− 0.03	− 0.07	− 0.09	− 0.09	0.53^***^	-		
8. T2 Fear of missing out	0.05	− 0.05	− 0.02	− 0.03	− 0.01	0.12^**^	0.17^***^	-	
9. T2 Short-form video addiction	− 0.03	− 0.02	− 0.02	− 0.05	0.00	0.23^***^	0.20^***^	0.42^***^	-
Mean	19.17	0.38	1.52	1.43	4.17	3.56	2.69	2.65	18.34
*SD*	0.80	0.49	0.78	0.78	1.32	0.96	0.83	0.91	7.51

Note. Gender:0 = male, 1 = female; T1 = Time 1; T2 = Time 2; ^*^p < 0.05, ^**^p < 0.01, ^***^p < 0.001.

### Testing the mediating role of fear of missing out

**Fig. 2:**
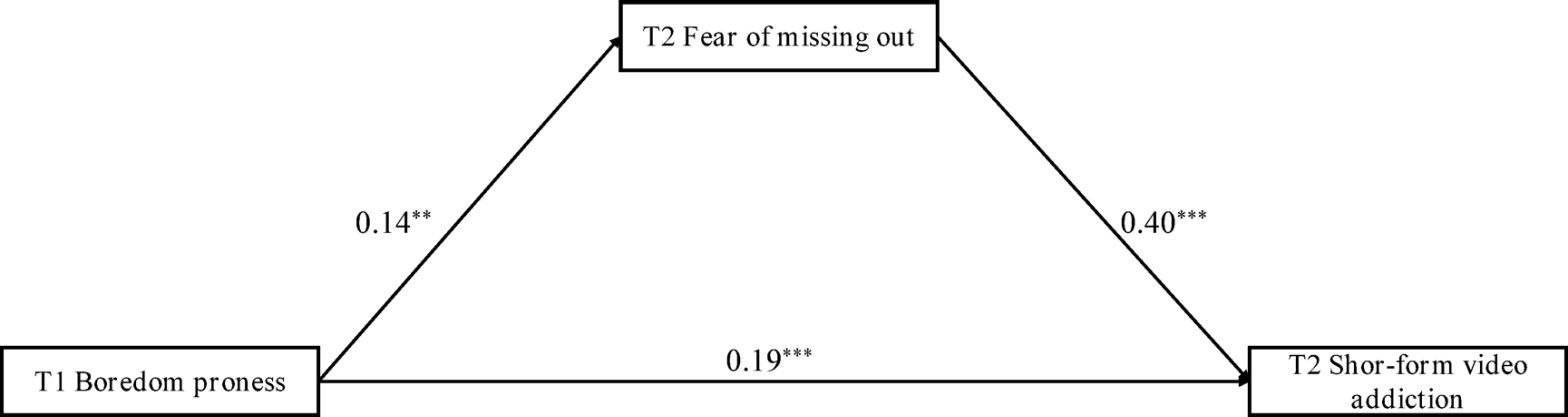
Standardized path coefficients for the mediation model of boredom proneness, fear of missing out, short-form video addiction; T1 = Time 1; T2 = Time 2; ^**^p < 0.01; ^***^p < 0.001.

The standardized path coefficients for the mediation model of boredom proneness, FoMO, and SFVA were shown in Fig. 2. As presented, T1 boredom proneness positively associated with T2 SFVA (*b* = 0.19, *SE* = 0.05, *p* < 0.001), T1 boredom proneness positively associated with T2 FoMO (*b* = 0.14, *SE* = 0.05, *p* < 0.01), and T2 FoMO positively associated with T2 SFVA (*b* = 0.40, *SE* = 0.04, *p* < 0.001). The bias corrected percentile bootstrap analyses shown that the indirect effects of T1 boredom proneness on T2 SFVA mediated by T2 FoMO was significant (indirect effect = 0.055, Boot *SE* = 0.026, 95% CI = [0.008, 0.108]).

### Testing the moderating role of intolerance of uncertainty

**Fig. 3:**
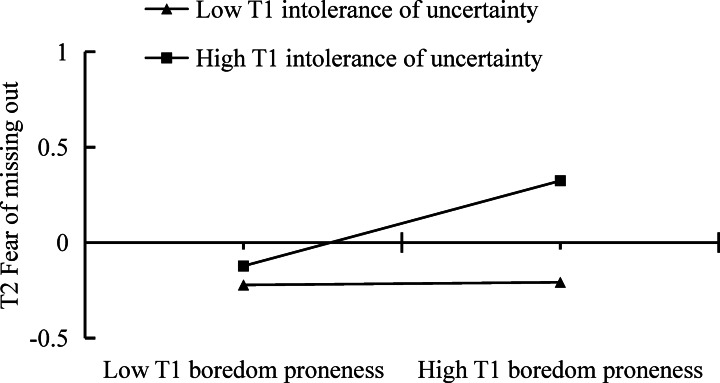
The moderating effect of intolerance of uncertainty on the association between boredom proneness and fear of missing out.

The standardized path coefficients for the moderated mediation model of boredom proneness, intolerance of uncertainty, FoMO, and SFVA were shown in Table 2. To avoid multicollinearity issues, all independent variables were standardized before constructing interaction terms. The results shown that the interaction term between boredom proneness and intolerance of uncertainty significantly associated with FoMO in Model 1 (*b* = 0.12, *SE* = 0.04, *p* < 0.01), indicating the moderating effect of intolerance of uncertainty. Using simple slope analysis, significant interactions both below and above 1 standard deviation (SD) from the mean of intolerance of uncertainty were demonstrated in Fig. 3. Specifically, T1 boredom proneness was associated with T2 FoMO only among college students with higher levels of T1 intolerance of uncertainty (*b* = 0.24, *p* < 0.01), but not did among college students with lower levels of T1 intolerance of uncertainty (*b* = 0.01, *p* = 0.903).

**Table 2:** Results of the moderated mediation model.

	Model 1: T2 FoMO		Model 3: T2 SFVA	
Predictor variable	*b*	SE	*b*	SE
Age	− 0.05	0.06	− 0.00	0.05
Gender	0.13	0.10	− 0.05	0.09
Father’s education level	0.00	0.07	0.00	0.07
Mother’s education level	− 0.03	0.07	− 0.04	0.07
Average monthly family income	0.01	0.04	0.02	0.03
T1 Boredom proneness	0.12^*^	0.06	0.19^***^	0.06
T1 IOU	0.16^**^	0.06		
T1 Boredom proneness × T1 IOU	0.12^**^	0.04		
T2 FoMO			0.40^***^	0.04
*R* ^*2*^	0.05		0.21	
*F*	3.16^**^		17.40^***^	

Note. Gender:0 = male, 1 = female; T1 = Time 1; T2 = Time 2; Fear of missing out = FoMO; Intolerance of uncertainty = = IOU; Short-form video addiction = SFVA; ^*^p < 0.05, ^**^p < 0.01, ^***^p < 0.001.

Furthermore, the mediating effect of T2 FoMO in the relation between T1 boredom proneness and T2 SFVA was conditioned by T1 intolerance of uncertainty (*b* = 0.05, *SE* = 0.02, 95% CI = [0.007, 0.085]). Specifically, the indirect effect of T2 FoMO was statistically significant only for college students with higher levels of T1 intolerance of uncertainty (indirect effect = 0.096, SE = 0.037, 95% CI = [0.022, 0.170]), but not did among college students with lower levels of T1 intolerance of uncertainty (indirect effect = 0.003, SE = 0.031, 95% CI = [−0.058, 0.065]).

## Discussion

Boredom proneness has been confirmed as a risk factor for mobile phone addiction among college students. However, the correlation between boredom proneness and their subsequent SFVA and its underlying psychological mechanisms remain inadequately understood. This study aims to addresses these research gaps by using a time-lagged research design with a moderated mediation model in Chinese college students. As expected, the results confirmed the mediating role of FoMO in the relations between boredom proneness and SFVA, and this association was moderated by intolerance of uncertainty.

### Linking boredom proneness and Short-form video addiction

The current results supported Hypothesis (1): Boredom proneness significantly associated with SFVA six months later, which was align with previous cross-sectional and longitudinal studies on mobile phone addiction and problematic mobile phone use^13,22^. Based on the findings of previous research and the current study, boredom proneness may be significantly associated not only with current SFVA among college students but also with the prospective development of SFVA six months later. On one hand, the frequent reward deficiency symptoms of individuals with a high tendency to be bored may reflect a dysfunction of the dopamine system, which makes them feel bored when exposed to routine stimuli and induces various addictive behaviours^47^. Short-form videos can automatically filter and push content based on personal preferences, quickly and inexpensively bringing a sense of pleasure and excitement, which is highly attractive to youth who are bored^9^. On the other hand, short-video platforms provide users with a sense of social identity by enabling them to follow and interact with others^1^. This social interaction function enables individuals with high level of boredom to rely on short-form video platforms for emotional comfort, thus escaping from social failures in the real world^17^.

Furthermore, the current findings supported the mediating role of FoMO between boredom proneness and SFVA (Hypothesis (2), which was consistent with previous studies^29,30^. In other words, individuals with high levels of boredom proneness are more likely to experience elevated levels of FoMO, which subsequently increases their risk of developing SFVA. From the perspective of the I-PACE model^16^, boredom proneness, as a personality trait, usually represents an individual’s strong desire for social information and novel stimuli. This dependence on external information also makes individuals with high boredom proneness more likely to experience fear, anxiety, and worry about information loss (i.e., FoMO)^29^. Considering that it is difficult to find matching stimulus information in offline life, short-from video platforms, as a fast-paced and low-cost entertainment and social space, are the convenient way to meet the needs of highly bored individuals. This forces them to spend a lot of time immersed in the online world, searching for potential solutions, which increases the risk of SFVA. Furthermore, individuals with higher FoMO may experience a depletion of cognitive resources due to their constant search for social information and interactions, which impairs their self-regulation and cognitive control^48^. This also makes individuals with high levels of FoMO vulnerable to SFVA.

This study addresses two main gaps. First, prior research has often treated boredom proneness as a mediating variable rather than as an independent predictor. Second, most existing studies have focused on general internet or mobile phone addiction, overlooking the unique mechanisms of SFVA. By positioning boredom proneness as an independent variable, this study offers novel evidence on its direct role in SFVA, thereby extending its theoretical significance. Moreover, by highlighting the distinct addictive patterns of SFVA, the findings provide new insights into the specificity of short-form video use beyond broader internet-related addictions.

### The roles of intolerance of uncertainty

This study found that intolerance of uncertainty exacerbated the association between boredom proneness and FoMO, thereby increasing the risk of SFVA among college students. Specifically, boredom proneness was significantly associated with an increase in FoMO over time, but this relations was significant only among college students with higher levels of intolerance of uncertainty. This result aligns with the prevailing view that individuals with high levels of intolerance of uncertainty are more susceptible to anxiety and restlessness when confronted with insufficient information^32,33^.

Individuals with high levels of intolerance of uncertainty exhibit heightened sensitivity to uncertainty and ambiguous situations, often perceiving them as threats^32^. This cognitive bias hinders individuals’ ability to manage uncertainty in their daily lives with rationality and calmness, they thus may feel intense anxiety and discomfort due to excessive focus on social and entertainment information (i.e., FoMO) that they may have missed^49^. Considering that individuals with a high boredom proneness often confront a lack of meaningful information and stimuli^15^, anxiety, worry, and restlessness with intolerance of uncertainty may persist and be difficult to overcome. Furthermore, the information dissatisfaction triggered by FoMO may drive people to seek online feedback on short-form video platforms. Within the framework of the I-PACE model^16^, boredom proneness can be regarded as a person-related vulnerability factor, intolerance of uncertainty as a maladaptive cognitive bias, and FoMO as a negative affective response. These processes interact to impair self-regulation at the execution stage, reinforcing short-form video use. People with a high level of intolerance of uncertainty are more sensitive to reward signals (such as likes, comments, and follows) on short-form video platforms, and can temporarily satisfy their anxiety and unease by monitoring online information and the social activities of others^48^. However, this sensitivity further deepens their dependence on short-form video platforms, perpetuating the cycle of online interactions and the search for rewards, and ultimately leading to SFVA.

Aligned with the I-PACE model, intolerance of uncertainty can be conceptualized as a cognitive vulnerability that interacts with affective predispositions, such as boredom proneness, to foster maladaptive digital media use. Boredom proneness heightens the need for external stimulation, while intolerance of uncertainty limits tolerance for ambiguity, together amplifying sensitivity to uncertain or novel cues and thereby intensifying FoMO. This pathway exemplifies the I-PACE proposition that affective and cognitive state jointly increase cue-reactivity, predisposing individuals to compulsive engagement with short-form video platforms.

In contrast to prior work that primarily emphasized the direct association between FoMO and social media use^26^, the present findings reveal how boredom proneness and intolerance of uncertainty interact to shape FoMO, thus clarifying mediating and moderating mechanisms underlying individual differences in short-form video addiction. By specifying how affective and cognitive vulnerabilities converge, this study refines the I-PACE framework and suggests that effective interventions should address not only FoMO but also upstream vulnerabilities such as boredom proneness and intolerance of uncertainty. Future research employing longitudinal or experimental designs could further illuminate causal pathways and explore additional mediators, including emotion regulation, self-control, and social support.

## Limitations

There are several limitations to this study. First, the data relied on self-reported measures, which may affect the internal validity of the findings. To reduce participants’ tendency to respond in socially desirable ways, future research could incorporate multiple methods or multiple informants for testing the stability of results. Second, most participants were college students, making the sample more representative of the young adult demographic. Future studies should broaden the age range to include adolescence and other stages of adults in the relationships between boredom proneness, FoMO, and SFVA. It may also be worth considering the relationship between different demographic characteristics of college students and the study variables, such as major and marital status. Finally, this study did not incorporate all variables in each wave of data collection. Future research could more thoroughly examine the relationships between these variables by incorporating all variables in each wave of data to explore the potential bidirectional relationships between boredom proneness, FoMO, and SFVA, as well as the moderating role of intolerance of uncertainty in these relationships.

## Conclusions

This study elucidated the potential mediating processes and conditional risk mechanisms underlying the relations between boredom proneness and SFVA among college students, highlighting the critical role of intolerance of uncertainty in addressing SFVA in this population. Results highlights the importance of timely interventions targeting college students’ experiences of boredom to prevent SFVA, such as through cognitive-behavioral therapy^49^. Furthermore, this study demonstrated that boredom proneness was significantly associated with FoMO only among college students with high levels of intolerance of uncertainty, and further associated with SFVA six months later. Therefore, for college students prone to boredom, interventions to prevent SFVA should consider addressing their intolerance of uncertainty. For instance, mindfulness-based cognitive therapy has been shown to be particularly effective in intervening in Intolerance of uncertainty^50^.

## Data Availability

The datasets generated and/or analysed during the current study are not publicly available due (our experimental team’s policy) but are available from the corresponding author on reasonable request.
